# How (Ir)rational Is it to Believe in Contradictory Conspiracy Theories?

**DOI:** 10.5964/ejop.v15i1.1690

**Published:** 2019-02-28

**Authors:** Petar Lukić, Iris Žeželj, Biljana Stanković

**Affiliations:** a Department of Psychology, Faculty of Philosophy, University of Belgrade, Belgrade, Serbia; b Laboratory for Social Psychology, Faculty of Philosophy, University of Belgrade, Belgrade, Serbia; Webster University Geneva, Geneva, Switzerland; University of Belgrade, Belgrade, Serbia

**Keywords:** conspiracy theories, irrationality, contradictory beliefs, overcoming contradictions

## Abstract

There is evidence that not only believing in one conspiracy theory (CT) makes a person more probable to believe in others, however unrelated their content is, but that people can even believe in contradictory CTs about a single event. After piloting locally relevant conspiracy theories on a convenient Serbian speaking sample (N = 152), we sought to replicate this finding on a larger sample (N = 252), but introduced several changes. We differentiated necessarily and probably mutually exclusive CTs, and interviewed the participants who answered contradictory to understand the reasoning behind it. The participants were more prone to endorse probably than necessarily exclusive items (we registered positive correlations in former and no correlation or negative correlation in later). Two strategies enabled them to overcome the contradiction: (a) distilling the crucial content and downplaying other information and (b) treating the contradictory scenarios as possible versions of events. Taken together, these results indicate that participants are not as irrational as sometimes portrayed.

Conspiracy theories (CTs) are narratives that offer an explanation of important social events. These narratives stand as an alternative to the official ones (governmental, academic, institutional, etc.), usually containing an assumption about a small group of malevolent, powerful actors, collaborating in secrecy. Albeit alternative, CTs are not endorsed by a neglectable minority of the population. On the contrary, for example, data show that around 50% of US citizens believe in at least one conspiracy theory regarding medical topics (Oliver & Wood, 2014a). One telephone survey showed that 41% of randomly selected participants believed that US Air Force is hiding evidence that the USA had been visited by UFOs and that 69% of the participants thought that it is likely that assassination of John F. Kennedy was secretly planned by powerful political actors (Goertzel, 1994). In a nationally representative survey of adult Serbian citizens, out of those who have heard about it, 44% agreed that HAARP system controls the weather, and 35% that moon landing was staged (Milošević-Đorđević & Žeželj, 2017). In addition, there is evidence that belief in CTs can lead to different behavioral outcomes: people who are more prone to believe in CTs are more likely to avoid the traditional medicine, less willing to get vaccinated (Oliver & Wood, 2014b), and to adhere to the prescribed therapy (Bogart, Wagner, Galvan, & Banks, 2010). CTs are also a common part of the political propaganda and are used as a mean of intimidation and manipulation of public opinion (Goertzel, 2010; Gray, 2008). Experimental studies showed that even mere exposure of CT to a person can make them less willing to vote, to engage in charity work or to sign a petition to stop global pollution (Douglas, Sutton, Jolley, & Wood, 2015; Jolley & Douglas, 2014; van der Linden, 2015).

While CTs can be construed around a wide range of social events, from the origin of HIV to the existence of UFO, they are usually positively interrelated. Goertzel (1994) showed that there is a general factor of believing in different conspiracy theories. In other words, if a person believes in one CT, he/she is more prone to believe in others, no matter how different their content is (Lukić & Žeželj, 2017; Oliver & Wood, 2014b; Swami, Chamorro‐Premuzic, & Furnham, 2010; Swami et al., 2011; van Prooijen, Krouwel, & Pollet, 2015; Wood, Douglas, & Sutton, 2012). In this system, labeled "monological belief system", one CT supports the others.

Furthermore, there is evidence that not only believing in one CT makes a person more prone to believe in other CTs about different events, but a person is also more likely to believe in a CT about *the same event* that contradicts it. In an influential study designed to test these assumptions, Wood et al. (2012) registered positive correlations between believing in mutually exclusive CTs (i.e. “(Princess) Diana faked her own death so that she and Dodi (Al-Fayed) could retreat into isolation”, “One or more rogue ‘cells’ in the British secret service constructed and carried out a plot to kill (princess) Diana” and “Business enemies of Dodi and his father Mohammed Al-Fayed assassinated Dodi, with the death of (princess) Diana a cover-up for the operation”). They explained this finding not by assuming there were multiple CTs in the system that support each other, but by assuming that each CT was in coherence with a higher-order belief (e.g. The official narratives are not true). One potential measure of such a more general, higher order belief, could be the so-called "conspiracy mentality" which is defined as “general propensity to subscribe to theories blaming a conspiracy of ill-intending individuals or groups for important societal phenomena” (Bruder, Haffke, Neave, Nouripanah, & Imhoff, 2013, p. 2); however, the authors did not include it in their study.

Even though Wood et al. (2012) were careful in explaining their central finding, it is often evoked in the academic debate to illustrate how irrational it is to believe in CTs (Brotherton, French, & Pickering, 2013; Swami, Voracek, Stieger, Tran, & Furnham, 2014; van der Linden, 2015). Even the proposed explanation about a higher order belief is contested, and instead it is proposed that the conspiracy believers are prone to so called “double think”, i.e. to simultaneously endorse contradictory claims (Irwin, Dagnall, & Drinkwater, 2015). We argue, however, that the obtained correlation could be an artifact of the way we typically pose questions in surveys, and that respondents could have interpreted it in a way that allowed them to circumvent contradiction. Having this in mind, we sought to replicate the initial finding, but we introduced several important changes in the design. First, we included a more general measure of conspiratorial mentality to study its relation to specific CTs. Second, we drew a distinction between the beliefs that are *necessarily* mutually exclusive (princess Diane can either be dead or alive), and the ones that are *probably* mutually exclusive, since they may simultaneously be true, although that is highly unlikely (princess Diane death could be conceived by an actor A or a B, but one can imagine a coherent narrative in which there could be a cooperation between these two actors). Although the authors did differentiate these types of relations between the CTs (compare Study 1 and Study 2, Wood et al., 2012), they did not contrast them in a single design. Third, to shed a light on the process of responding and the underlying reasoning, upon finishing the survey, we recruited and interviewed participants who endorsed contradictory CTs.

The aim of the present study was (a) to explore if endorsement of different CTs is positively correlated and correlated to conspiracy mentality (b) to specifically analyze the correlation between contradictory CTs, and compare the ones necessarily exclusive and probably exclusive, (c) to register and categorize the cognitive strategies respondents use when endorsing contradictory statements.

We expected that endorsement of different specific CTs would correlate positively (H1) and that it would be correlated with general proneness to conspiratorial thinking (H2). Furthermore, we expected that endorsement of contradictory CTs would correlate positively (H3), although the participants would be more prone to simultaneously endorse pairs of probably exclusive than pairs of necessarily exclusive CTs (H4).

## Method

### Participants and Procedure

To pre-select familiar CTs, we conducted an online pilot survey (number of participants *N* = 152, 65 males) with 32 items containing potentially widespread CTs. We constructed the scale drawing from previous published international works (Goertzel, 1994; Swami et al., 2010; Brotherton et al., 2013; Dagnall, Drinkwater, Parker, Denovan, & Parton, 2015), and scarce regional ones (Blanuša, 2009, 2011), as well from the traditional media, social networks, forums and blogs. For the final version, we selected items with the average familiarity of at least three on a five-point scale.

After the pilot study that allowed us to construct a culturally sensitive instrument for assessing belief in CTs, we conducted the main study on a different sample. The participants were 252 high school and university students (82 males, *M*_Age_ = 19.77, *SD* = 1.58). We chose this particular age group (late adolescents and emerging adults), as they are still in process of forming a coherent worldview, so we reasoned they might be more prone to endorsing contradictory narratives. They completed a pen and pencil survey under a self-generated code name.

Upon analyzing the data, we formed a subsample of participants who endorsed contradictory CTs by answering with either 3 - *Agree* or 4 – *Strongly agree* on a four-point Likert scale (*N* = 122). Out of those, we randomly polled 26 participants and conducted semi-structured interviews with them^i^. Interviews were recorded and transcribed verbatim for further analysis. The sampling for this qualitative phase of the study was ceased when the theoretical saturation was reached.

### Instruments

Belief in Specific CTs. We designed a 16-items questionnaire covering various CTs chosen in the pilot stage (i.e. “1969 moon landing of US astronauts was staged”; "Pharmaceutical companies deliberately keep the cure for HIV away from the public"). Items were evaluated using a Likert scale with a 4-point response format (1 = *Completely disagree*, 4 = *Completely agree*). We excluded the midpoint of the Likert type scale used to measure the endorsement of CTs, thereby forcing the respondents to decide whether it is more likely to be true or not. We reasoned that having a midpoint might have artificially inflated the correlations in the initial study, as they might have partially stemmed not from endorsing the CTs but from being indecisive. Internal consistency (Cronbach's Alpha = .78) allowed us to compute a single score.

Contradictory CTs. On top of chosen 16, we added nine mutually contradictory items, out of which some were necessarily exclusive (i.e. “Slobodan Milošević was killed in the Hague” and “Slobodan Milošević did not actually die in the Hague, but faked his own death”) and some probably exclusive (i.e. “HIV virus is deliberately produced in the USA” and “HIV virus is deliberately produced in Europe”). To avoid circularity in later correlational analysis, these items were not included in the total score of Belief in Specific CTs. Locally specific contradictory CTs were construed around three events: the death of Slobodan Milošević in the Hague, the assassination of Željko Ražnatović Arkan and the true identity of Josip Broz Tito. Slobodan Milošević (1941–2006) was a president of Serbia during the wars in Croatia and Bosnia in the 1990s. He was charged with war crimes by the International Criminal Tribunal for the Former Yugoslavia in the Hague. While the process against him was still ongoing, he died of natural causes in the Hague prison. There were several alternative accounts to his death in the Serbian public: one assumed he was still alive and living in Russia, whilst the other assumed he did not die of natural causes but was murdered in the Hague. Željko Ražnatović Arkan (1952–2000) was a commander of paramilitary forces in the Yugoslav wars and a habitual offender wanted by the Interpol. He was nevertheless politically active during the war years (1990s) in Serbia. Arkan was killed in a hotel lobby in 2000. Even though the shooter was charged and prosecuted, the political motives behind the murder, as well as the suspicion that he is in fact still alive, are still discussed in the official and social media. Josip Broz Tito (1892–1980) was a Yugoslav communist revolutionary leader, serving as a first president of Socialist Federal Republic of Yugoslavia for more than 30 years. In Serbia, but also in all ex-Yugoslav countries, there are multiple CTs regarding his origin, identity and political past, partly due to his skillful equilibrating between East and West during the Cold war.

To measure proneness to conspiratorial thinking, we adapted five-item Conspiracy mentality questionnaire (CMQ) constructed by Bruder et al. (2013) with a 5-point response format (1 = *Completely disagree*, 5 = *Completely agree*).

Semi-structured interview consisted of several parts. The first part aimed at warming up the participants and leading them to think through their answers on the questionnaire (“Do you remember your answer to the item ‘1969 moon landing of US astronauts was staged?’” and “Why did you answer like that?”). Next part consisted of the same questions as the previous one, but this time concerning a pair of mutually exclusive items that the participant endorsed. Following that, we pointed to the fact that this is a case of contradictory claims and asked the participant to give us the reason behind endorsing both (“One might say that this representation of the event and the previous one can’t go together, that they contradict each other. How do you understand this? Can you explain how both can be true?”). This procedure has been repeated with all pairs of contradictory items endorsed by the participant.

## Results

We will start with a detailed description of CM and specific CT endorsement in the sample, and then proceed to test the hypotheses about their interrelatedness, as well as the endorsement of contradictory CTs of different types. Finally, we will qualitatively analyze the strategies respondents reported to have used when they endorsed contradictory narratives and illustrate them with typical citations by the participants. The instruments, database and interview transcripts are available at https://osf.io/zxq2x/.

The reliability of Belief in Specific CTs questionnaire was α = .78, and the reliability of CMQ on our sample was α = .67, slightly lower than in the original study (reliability of different versions of the questionnaire ranged from α = .72 to α = .84, Bruder et al., 2013). The exploratory factor analysis yielded with one factor explaining 44% of the variance. Taken together, this allowed us to calculate a summary score. The average endorsement, standard deviation and the percentage of endorsement for all CTs used in present research are detailed in Table 1.

**Table 1:** Mean, Standard Deviation and Percentage of Endorsement of CMQ and Specific CTs

Items	*M*	*SD*	%
Conspiracy Mentality Questionnaire			
Politicians usually do not tell us the true motives for their decisions.	4.57	0.71	91.30%
Many very important things happen in the world, which the public is never informed about.	4.47	0.82	89.72%
There are secret organizations that greatly influence political decisions.	4.23	0.91	77.86%
Events which superficially seem to lack a connection are often the result of secret activities.	3.92	0.95	69.56%
Government agencies closely monitor all citizens.	3.45	0.98	47.43%
Specific CTs			
Some of the ingredients of Coca-Cola should produce an addiction in its consumers.	3.42	0.78	87.35%
Certain commercials on television contain subliminal messages which negatively affect our behavior without our knowledge.	3.18	0.88	80.23%
Trails in the sky that are left behind the airplane contain chemicals which pollute the living environment and poison people.	2.76	0.90	63.24%
Pharmaceutical corporations are keeping the cure for HIV in secret.	2.74	1.04	61.66%
Pharmaceutical corporations sell vaccines of poor quality on the Eastern-European market.	2.68	0.85	57.70%
Freemasons is a secret organization that controls the world from the shadow.	2.68	1.00	56.52%
Željko Ražnatović Arkan was killed by the secret security service.^a^	2.67	0.96	57.70%
It is possible to control the weather conditions by using HAARP.	2.61	0.99	56.91%
HIV is deliberately made in the USA laboratories to reduce the percentage of black population in Africa.^a^	2.56	1.00	51.77%
The fact that the vaccines are bad for children’s health is kept away from public.	2.54	1.01	50.59%
Slobodan Milošević did not die by natural death, but was murdered in the Hague.^a^	2.54	0.98	52.96%
The attack on the World Trade Centre in New York in 2001 is performed by the USA secret services.	2.52	1.00	49.80%
Pharmaceutical corporations use citizens of Eastern-European countries as guinea pigs for testing new medications.	2.50	0.91	50.98%
The 1969 moon landing of American astronauts was staged.	2.46	1.05	46.64%
Pharmaceutical corporations use vaccines in order to cause certain diseases in people.	2.39	0.97	43.08%
Josip Broz Tito was a high ranked member of Freemasons.	2.30	0.92	39.52%
HIV is deliberately made in the laboratories of European pharmaceutical corporations to reduce African population and thus to control the flow of immigrants from Africa into Europe.^a^	2.24	0.96	36.75%
Proofs of alien existence are kept away from public.	2.17	1.06	37.15%
The death of Adolf Hitler was staged.	2.13	1.03	35.96%
HIV is deliberately made in laboratories of Central Africa.^a^	2.13	0.99	35.17%
Željko Ražnatović Arkan faked his death.^a^	1.90	0.95	24.11%
Josip Broz Tito was an American spy.^a^	1.76	0.85	15.41%
The real Josip Broz Tito died during WWII and was then replaced by a double.	1.71	0.92	17.39%
Tito was a Russian spy.^a^	1.61	0.73	9.09%
Slobodan Milošević did not die in the Hague, but faked his death.^a^	1.41	0.66	4.74%

^a^Mutually exclusive items.

Measurement model. To examine the factor structure of the specific conspiracy theories questionnaire, the 16 items were subjected to an exploratory factor analysis. The results corroborated our first hypothesis about the interrelatedness between CTs of very different content. Both parallel and scree criteria indicated a one-factor solution. The size of the Kaiser-Meyer-Olkin measure of sampling adequacy, KMO = .78, and significance of Bartlett’s test of sphericity (χ^2^ = 671.71, *p* < .001) showed that the 16 items had adequate common variance for principal component analysis. The number of components to be extracted was determined by parallel criteria (Horn, 1965) and inspection of the scree plot (Cattell, 1966; Buja & Eyuboglu, 1992). Based on the mentioned criteria, one factor emerged with parallel criteria average higher than the 95^th^ percentile, explaining 24% of total variance, with item loadings varying from .32 (“Hidden messages in commercials”) to .62 (“Vaccines cause diseases”). To test the assumption that items from Belief in Specific CTs questionnaire and Conspiracy mentality questionnaire will form two separate factors, we compared a single-factor model and a two-factor model in confirmatory factor analysis. We found that two-factor model (χ^2^/*df* = 1.98, GFI = .88, RMSEA = .06, CFI = .79), although not optimal on all parameters, fitted the data better than single-factor model (χ^2^/*df* = 2.25, GFI = .82, RMSEA = .07, CFI = .73).

Full correlation matrix of specific CTs and CMQ items is detailed in Table 2. Specific CTs and CMQ items were either positively related or unrelated. Similarly, all pairs of contradictory items were mostly related to other CTs and items from CMQ, forming an expected pattern. Also in line with initial expectations, we registered a substantial correlation between average specific CT endorsement and CM (*r* = .49, *p* < .001).

**Table 2a:** Intercorrelations of Endorsements of Items From Belief in Specific CTs Questionnaire and Conspiracy Mentality Questionnaire, Part 1

Items	1	2	3	4	5	6	7	8	9	10	11	12	13	14	15
1. Subliminal messages	–	.11	.05	.07	.06	.13*	.27**	.10	.05	.20**	.12	.14*	.11	.21**	.12*
2. Tito Freemason		–	.26**	.24**	.19**	.27**	.11	.09	.08	.11	.23**	.14*	.28**	.01	.32**
3. Tito’s double			–	.17**	.25**	.22**	.02	.20**	.05	.09	.04	.11	.13*	.17**	.29**
4. Moon landing				–	.35**	.16*	.12	.27**	.22**	.19**	.28**	.25**	.32**	.16*	.19**
5. Hitler faked his death					–	.18**	.06	.17**	.10	.17	.19**	.14*	.29**	.06	.01
6. HAARP						–	.21**	.29**	.19**	.25**	.31**	.13*	.24**	.18**	.30**
7. Coca-Cola							–	.09	.17**	.18**	.25**	.18**	.16*	.09	.19**
8. HIV cure hidden								–	.26**	.33**	.39**	.25**	.32**	.12	.26**
9. Vaccines East Europe									–	.31**	.22**	.34**	.25**	.16*	.15*
10. Vaccines harmful										–	.42**	.16*	.20**	.24**	.23**
11. Vaccines diseases											–	.22**	.35**	.17**	.13*
12. Guinea pigs												–	.17**	.27**	.28**
13. 9/11													–	.08	.08
14. Chemtrails														–	.16
15. Freemasons															–
16. Aliens proof hidden															
17. HIV made in USA															
18. HIV made in Europe															
19. HIV made in Africa															
20. Tito Russian spy															
21. Tito American spy															
22. Arkan murdered															
23. Arkan alive															
24. Milošević murdered															
25. Milošević alive															
26. Public not informed															
27. Motives of politicians															
28. Agencies monitoring															
29. Unrelated events															
30. Secret organizations															

**Table 2b:** Intercorrelations of Endorsements of Items From Belief in Specific CTs Questionnaire and Conspiracy Mentality Questionnaire, Part 2

Items	16	17	18	19	20	21	22	23	24	25	26	27	28	29	30
1. Subliminal messages	.14*	.19**	.14*	.06	.05	.04	.15*	.09	.12	-.03	.30**	.19**	.17**	.17**	.29**
2. Tito Freemason	.18**	.23**	.19**	.20**	.24**	.23**	.10	.01	.21**	.04	.06	-.02	.07	.15*	.21**
3. Tito’s double	.14*	.09	.08	.01	.13*	.25**	.10	.28**	.25**	.20**	-.04	-.03	.11	-.05	.19**
4. Moon landing	.24**	.29**	.12	.12	.09	.17**	.16*	.11	.27**	.07	.12	.09	.06	.15*	.24**
5. Hitler faked his death	.21**	.17**	.02	.19**	.14*	.17**	.13*	.19**	.22**	.15*	.10	.08	.07	.14*	.15*
6. HAARP	.21**	.23**	.17**	.22**	.13*	.11	.16*	.15*	.22**	.10	.16*	.06	.18**	.05	.24**
7. Coca-Cola	.10	.21**	.08	.18**	-.04	.07	.19*	.04	.18	-.01	.11	.10	.16**	.09	.21**
8. HIV cure hidden	.13*	.41**	.36**	.22**	.07	.19**	.13*	.08	.14*	.14*	.21**	.07	.15*	.13*	.33**
9. Vaccines East Europe	.17**	.32**	.04	.11	.15*	.13*	.19**	.03	.17**	.04	.09	.28**	.11	.22**	.25**
10. Vaccines harmful	.17**	.25**	.17**	.08	.07	.21**	.29**	.03	.14*	.15*	.21**	.08	.20**	.17**	.39**
11. Vaccines diseases	.13*	.35**	.22**	.29**	.08	.13*	.20**	-.02	.16*	.14*	.16**	.14*	.17*	.21**	.29**
12. Guinea pigs	.08	.25**	.11	.07	.05	.08	.17**	.07	.30**	.06	.17**	.14*	.16*	.29**	.28**
13. 9/11	.11	.27**	.21**	.31**	.13*	.14*	.02	.13*	.17**	.02	.09	.14*	.12	.17**	.30**
14. Chemtrails	.22**	.14*	.08	.05	.10	.12	.16*	.08	.17**	.12	.19**	.12	.09	.12	.26**
15. Freemasons	.17*	.22**	.20**	-.02	.11	.16*	.19**	.10	.25**	.10	.12	.02	.20**	.12	.38**
16. Aliens proof hidden	–	.12	.13*	.09	.14*	.12	.13*	.20**	.16*	.04	.12	.09	.18**	.15*	.20**
17. HIV made in USA		–	.58**	.28**	.06	.12	.17**	.05	.18**	.04	.23**	.10	.01	.21**	.32**
18. HIV made in Europe			–	.17**	.13*	.03	.01	.16*	.05	.10	.17**	-.01	.07	.16*	.18**
19. HIV made in Africa				–	.12*	.03	.15*	-.02	.10	.06	.06	.04	-.04	.17**	.12
20.Tito Russian spy					–	.23**	.11	.01	.08	.17**	.02	.04	.05	.13*	.06
21. Tito American spy						–	.07	.20**	.16*	.37**	.03	-.04	.03	.03	.14*
22. Arkan murdered							–	-.27**	.31**	-.01	.14*	.02	.11	.15*	.23**
23. Arkan alive								–	.01	.32**	.05	.05	.06	.03	.04
24. Milošević murdered									–	.06	.07	.08	.19**	.14*	.17**
25. Milošević alive										–	-.13*	-.09	-.13*	.01	-.08
26. Public not informed											–	.36**	.14*	.29**	.47**
27. Motives of politicians												–	.25**	.30*	.33**
28. Agencies monitoring													–	.26**	.27**
29. Unrelated events														–	.34**
30. Secret organizations															–

Endorsing contradictory CTs. Almost half of our participants (48.4%) endorsed at least one pair of mutually exclusive CTs. Participants endorsed more pairs of probably exclusive CTs than pairs of necessarily exclusive CTs (Table 3).

**Table 3:** Frequencies of Contradictory and Non-Contradictory Answers on Pairs of Probably and Necessarily Exclusive CTs

Type of answers	Probably exclusive CTs		Necessarily exclusive CTs	
	Origin of HIV	Tito	Arkan	Milošević
Contradictory answers	108 (42%)	6 (2%)	23 (9%)	6 (2%)
Non-contradictory answers	144 (58%)	246 (98%)	229 (91%)	246 (98%)

Unlike Wood et al. (2012), we found both positive and negative correlations between mutually exclusive CTs depending on the type of relation between them. Namely, probably exclusive CTs were positively correlated - specifically “HIV was produced in Africa”, “HIV was produced in Europe”, “HIV was produced in the USA” (*r*_USA-EUR_ = .58, *p* < .001; *r*_USA-AFR_ = .28, *p* < .001; *r*_EUR-AFR_ = .17, *p* < .001) and “Josip Broz Tito was a Russian spy” and “Josip Broz Tito was an American spy” (*r* = .23, *p* < .001). On the other hand, one pair of necessarily exclusive CTs was negatively correlated - “Željko Ražnatović Arkan was killed” and “Željko Ražnatović Arkan is still alive” (*r* = -.27, *p* < .001), whilst the other pair was unrelated - “Slobodan Milošević was killed in the Hague” and “Slobodan Milošević did not actually die in the Hague” (*r* = .06, *p* = .346)^ii^.

There were positive correlations between the number of endorsed pairs of mutually exclusive CTs and the score on Belief in Specific CTs questionnaire (*τ = .*31, *p <* .001; ρ = .37, *p <* .001)^iii^, as well as between the number of endorsed pairs of mutually exclusive CTs and the score on CMQ (*τ = .*20, *p <* .001; ρ = .24, *p <* .001).

Content analysis of the interview transcripts revealed two types of reasoning behind endorsing mutually exclusive items, upon we excluded cases of answering in a random or inattentive manner^iv^. All explanations given by the participants were unproblematically recognized as indicating one of the following strategies behind endorsing contradictory CTs:

a) *Distilling crucial content*. Emphasizing the shared aspect of contradictory items that is considered crucial and disregarding the aspect that is different because it is considered to be of secondary relevance. For example, the participants are more focused on the fact that HIV was man-made than on the actual geographical origin of the virus:

“It is irrelevant. I don’t know if it was produced in the USA or Europe, but I believe it was created deliberately, with a specific aim.”

“I truly believe it is fabricated intentionally! I wouldn’t bet on a particular place of origin. What is important to me is that it is created by someone, and not the details, if it was in Africa or Europe...”

It seems that the respondents are able to circumvent the contradiction by reinterpreting the claims and extracting the shared aspect of the content that is relevant to them – in this case, it is the thesis of a secret plot to create HIV virus. The concrete location is considered to be information of secondary importance and the one they are unsure of. Some participants even represent Europe and the USA as a single political area with shared interests (“Europe, America... it’s the Western civilization”), which further makes the difference in geographical origin of the virus irrelevant when endorsing the claims.

This first explanatory strategy was typical for probably exclusive CT items – 75% of the answers in this category were explained this way (and none of the necessarily exclusive CT items).

b) *Evoking possible scenarios.* Treating contradictory items as possible scenarios – different explanations that make sense, sound credible or could be accurate under certain conditions. Since participants are not sure which one of the alternative explanations is true, they endorse both as equally plausible:

“Well, I don’t know, either he was killed, or he staged his own death... For me, these are both possible options.”

“Well, it is possible that he is alive, but, in case he is dead, it certainly had something to do with the state.”

We could not assume that the respondents in this situation believe in any of the two options expressed by the contradictory claims, but they certainly suspect the official story about the event. So, they are not sure if someone was killed or faked his own death, but they are sure “there is something suspicious about it”. And that is why they endorse both claims as potentially true, although they do not know which of them actually happened. Therefore, they also evade contradiction since they endorse claims as “either A or B is true”, not as “both A and B are true”.

This second explanatory strategy was typical or necessarily exclusive CT items – all of the answers in this category were explained this way (and only 25% of the probably exclusive CT items).

## Conclusions and Implications

First, our results strongly corroborate the idea of CTs of very different content being related and forming a unified belief system. Most of the endorsements of specific CTs were moderately positively correlated and they form one principal component. We found a moderate positive correlation between belief in specific CTs and proneness to conspiratorial thinking, which goes in line with the results of previous research. Along with the results of confirmatory factor analysis that favor the two independent factors model, we argue that belief in specific CTs and conspiracy mentality, although correlated are not fully overlapping constructs. Namely, CMs content is generic and independent of cultural or historical content; due to abstractness of its items it can be more acceptable to general public in comparison to specific CTs – some of the CMQ items were even criticized to be factual, instead of conspiratorial (Swami et al., 2017).

Second, in their seminal study, Wood et al. (2012), reported only positive correlations between endorsements of contradictory CTs and did not differentiate between probably exclusive and necessarily exclusive CTs in a single design. In the present research, however, participants were sensitive to the type of mutual exclusiveness and the percentage of the endorsement of necessarily and probably exclusive items as well as their correlation support this conclusion. Around half of our participants endorsed at least one pair of contradictory CTs and, as predicted, participants endorsed pairs of probably exclusive items more than they endorsed pairs of necessarily exclusive ones. In addition, we registered positive correlations between CTs which were probably exclusive and no correlation or even negative correlation between those that we proposed as necessarily exclusive. This finding illustrates that it is important to further explore the logical relations between the beliefs and the way the participants organize them in coherent belief systems.

Third, through the interviews, we sought to understand the reasoning behind the endorsement of contradictory items. Analysis of the interview data indicates that in case of the agreement with contradictory CTs, the respondents actually interpret the claims so as to avoid contradictions. They construct a higher order interpretation of the events in which suspicion towards the “official story” is central and mutually exclusive CTs are seen as probable alternative explanations – only one of which might be true. This is consistent with the finding of Harambam and Aupers (2015) that people who believe in conspiracy theories perceive themselves as “skeptics”.

The qualitative analysis thus highlighted several problems with the questionnaire as a way to assess beliefs in CTs and the typical interpretations of questionnaire data. Namely, one concrete CT can be an elaborate system of beliefs which we simplify and narrow down to one sentence in a questionnaire. That forces the respondents to compromise when assessing their truthfulness. They reinterpret the statement in the way it fits their belief system and the information available to them, ignoring some pieces and cherry-picking other pieces of the CTs content in the process.

In addition, a participant’s agreement with a statement sometimes does not indicate the endorsement of its content, but the endorsement of any explanation that is alternative to the official one. The respondents assume that they have only a limited knowledge about certain events so they are not in a position to give definitive answers – as one of them said: “I can’t really claim something like that (regarding what happened in the case of Arkan’s murder), because I am not an important person". They actually respond to the items by assessing the probability of the given scenario – “There is a possibility that they did (landed on the Moon in 1969), but I can’t say ‘yes’ or ‘no’”. Since the respondents reported having assessed *probability* for an explanation to be true (it may have happened like that), this allowed for different (even contradictory) representations of events to be simultaneously probable, therefore contradictory beliefs to coexist without being irrational. Our findings can fit the three-level model recently put forward by Wood (2017), in which general conspiracy ideation leads to conspiracy suspicions about specific event, which in turn can breed different specific conspiracy beliefs. If the conspiracy beliefs can share a common assumption, however vague (e.g. “climate change/death of an important person is a hoax”), it is possible for them to simultaneously exist.

Taking all this into account, one should be careful before drawing a conclusion that the respondents who endorsed a CT really “believe” in it. Thus, participants’ answers on response scales should be interpreted more carefully. Additionally, more elaborate response scales could be developed that would allow e.g. separate assessment of the likelihood of an event or the credibility of the explanation etc., so the respondents’ relation towards the items becomes clearer. The knowledge about an event, i.e. the quantity and quality of the information available to a participant should also be assessed and its relationship with believing in CTs clarified.

On the other hand, even when they believe in a certain CT, their explanations indicate that this belief is in most cases really superficial – without any greater engagement in form of researching and considering facts that could support or discredit their belief. This is not surprising, since this research, as all the previous ones, has been conducted on the general population. Studying persons who are particularly committed to and involved with CTs could provide a better-grounded basis for drawing conclusions about reasoning behind beliefs in CTs, including those contradictory ones.

This research goes to show that people who endorse CTs are not as irrational as sometimes portrayed. Adequately understanding proneness to conspiracy beliefs is especially relevant in a contemporary context, characterized by complexity and ambiguity of social events and the abundance of information sources together with the lack of acknowledged epistemic authorities. Discussed methodological challenges indicate that conspiratorial worldview should be studied more thoroughly, preferably in the multi-method framework and with more sensitivity to its content and the participants’ responding strategies.

## Data Availability

For this study, a dataset is freely available (see the Supplementary Materials section).

## Notes

The instruments, database and interview transcripts for the present paper are available via the OSF project page (https://osf.io/zxq2x/).
